# Clinical comparison of patient outcomes following implantation of trifocal or bifocal intraocular lenses: a systematic review and meta-analysis

**DOI:** 10.1038/srep45337

**Published:** 2017-03-28

**Authors:** Zeren Shen, Yuchen Lin, Yanan Zhu, Xin Liu, Jie Yan, Ke Yao

**Affiliations:** 1 Eye Center, Second Affiliated Hospital, School of Medicine, Zhejiang University, Hangzhou, China

## Abstract

To assess the visual effects of trifocal intraocular lenses (IOLs) compared to bifocal IOLs in cataract surgery, a meta-analysis of prospective comparative clinical trials (including 4 randomized controlled trials and 4 cohorts) was conducted. The defocus curves showed a better distance-corrected intermediate visual acuity (VA) for the trifocal group (MD −0.07; 95% CI, −0.10 to −0.05; *p* < 0.00001), while the VA outcomes showed no significant difference in distance VA (MD −0.03; 95% CI, −0.06 to 0.01; *p* = 0.13 for uncorrected distance VA and MD −0.00; 95% CI, −0.01 to 0.01; *p* = 0.78 for distance-corrected distance VA), near VA (MD −0.01; 95% CI, −0.07 to 0.04; *p* = 0.68 for uncorrected near VA and MD −0.01; 95% CI, −0.06 to 0.04; *p* = 0.66 for distance-corrected near VA) or refraction between the two groups. Contrast sensitivity and subjective visual quality yielded less conclusive results. Overall, a patient may achieve better intermediate VA with a trifocal IOL than with a bifocal IOL without any adverse effect on distance or near VA. The findings on contrast sensitivity and subjective visual quality were heterogeneous, with no clear results favoring either option.

Cataracts are very common in older people, leading to a decrease in vision and quality of life. Surgery to remove the cloudy crystalline lens and replace it with an artificial intraocular lens (IOL) is the only effective treatment. More and more patients presenting for cataract surgery want to enjoy good vision at distance, intermediate, and near ranges without the use of spectacles. Multifocal IOLs that can provide a wide range of clear vision attempt to meet this objective^1^,^2^.

At present, most multifocal IOLs are bifocal, with only near and far foci; the quality of intermediate viewing activities such as computer use might be insufficient for daily life^3^,^4^,^5^. Manufacturers have recently introduced multifocal IOLs that are trifocal in design, providing functional far, intermediate, and near vision^2^,^6^,^7^,^8^. Optical evaluation of multifocal IOLs has demonstrated that trifocal IOLs achieved a useful third focus for intermediate vision but were associated with increased background glare and halos and reduced visual quality^9^,^10^.

Clinical evaluation of multifocal IOLs is less clear-cut. There have been several studies comparing visual outcomes after the implantation of bifocal and trifocal IOLs in recent years^1^,^2^,^3^,^4^,^5^,^6^,^7^,^8^. Some studies support the notion of trifocal IOLs as the next generation of multifocal IOLs, which improve intermediate vision and the continuum of functional vision without impairing distance and near vision^5^,^6^,^7^. However, another study reports that bifocal IOLs provide intermediate visual acuity (VA) similar to trifocal IOLs^2^. Other studies suggest that the diffractive design of trifocal IOLs splits more incoming light than bifocal IOLs, which has the potential to decrease contrast sensitivity and increase photic phenomena^1^,^9^. To our knowledge, no systematic review and meta-analysis has been reported on this topic. We sought to conduct a meta-analysis of the existing randomized controlled trials (RCTs) and prospective cohorts to compare the visual results achieved with trifocal IOLs and bifocal IOLs.

## Results

### Search results

**Figure 1:**
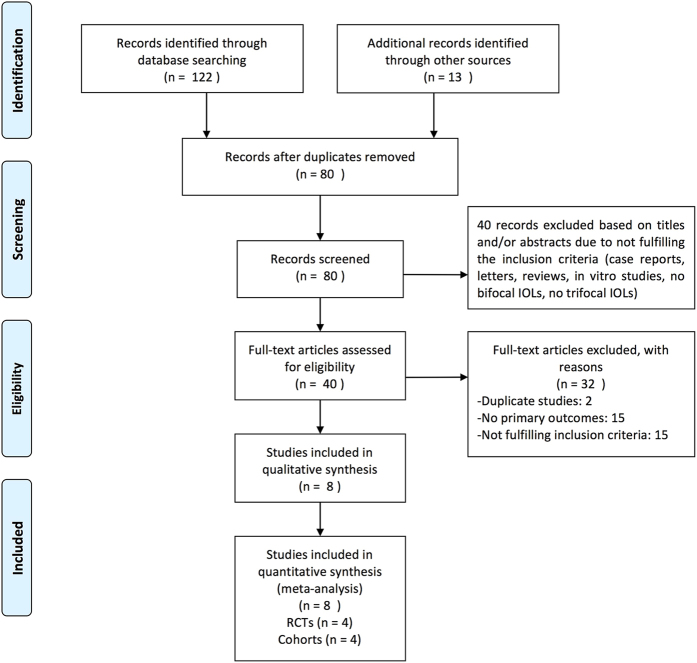
Flow chart showing selection of articles. IOL = intraocular lens; RCTs = randomized controlled trials.

After adjusting for duplicates, 80 different studies were identified. Of these studies, 40 were excluded because their titles or abstracts did not meet the inclusion criteria. The full text of each of the remaining 40 citations was examined in more detail. From these 40 citations, 32 studies were excluded for the following reasons: 15 did not fulfill inclusion criteria, 15 studies did not provide primary outcomes, and two were duplications. Four RCTs^2^,^3^,^4^,^5^ and four prospective cohorts^1^,^6^,^7^,^8^ were ultimately included in this meta-analysis. Figure 1 shows the flow diagram for the search and selection process.

### Study characteristics and quality

Eight studies that reported on 245 eyes (123 participants) with trifocal IOL implantation and 244 eyes (122 participants) with bifocal IOL implantation were included in this research. The eight studies were all conducted in European countries: France^4^, Norway^1^,^5^, the Netherlands^2^, the Czech Republic^6^, and Spain^3^,^7^,^8^. Table 1 summarizes the main characteristics of the included studies, and their quality is assessed in Supplementary Tables S1 and S2. Follow-up ranged from three to six months. Masking of surgeons is impossible in RCTs, and one study reported that patients were masked^2^. No RCT scored higher than three points. Of the prospective non-randomized comparative studies, one matched the preoperative VA of eyes in trifocal and bifocal groups^7^, two studies did not match preoperative VA^6^,^8^, and the other study did not discuss preoperative VA at all^1^. The age factor differed significantly between groups in two studies^1^,^6^. All four cohorts were of relatively low risk of bias, scoring equal to 7/8 on the Newcastle-Ottawa Scale (NOS). The overall quality of the meta-analysis is shown in Table 2. The assessment was considered to be of high to very low quality. Study design was the main reason to downgrade the overall quality of evidence, as the Grading of Recommendations Assessment, Development, and Evaluation (GRADE) group suggested. Moreover, high heterogeneity and a limited number of eyes enrolled downgraded the quality of outcomes.

### Primary outcome criteria

#### Visual acuity

**Figure 2:**
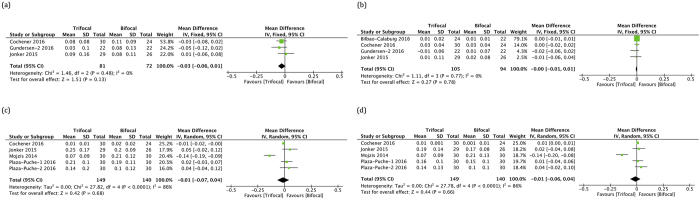
Pooled mean differences (MDs) for uncorrected distance visual acuity (UDVA), corrected distance visual acuity (CDVA), uncorrected near visual acuity (UNVA) and distance-corrected near visual acuity (DCNVA) in logMAR by meta-analysis. (**a**) Forest plot showing the MD of UDVA comparing trifocal intraocular lens (IOL) with bifocal IOL postoperatively (only for RCTs). (**b**) Forest plot showing MD of CDVA comparing trifocal IOL with bifocal IOL postoperatively (only for RCTs). (**c**) Forest plot showing the MD of UNVA comparing trifocal IOL with bifocal IOL postoperatively. (**d**) Forest plot showing the MD of DCNVA comparing trifocal IOL with bifocal IOL postoperatively.

Four RCTs reported uncorrected distance visual acuity (UDVA) or corrected distance visual acuity (CDVA) as outcomes (Fig. 2a and b, Table 2, Supplementary Table S3) and analyses including only RCTs did not reveal any statistically difference between the trifocal and bifocal groups (MD −0.03; 95% CI, −0.06 to 0.01; *p* = 0.13 for UDVA and MD −0.00; 95% CI, −0.01 to 0.01; *p* = 0.78 for CDVA)^2^,^3^,^4^,^5^. The quality of the evidence was moderate to high. Combined with cohorts, most studies reported these outcomes (Supplementary Figs S1 and S2)^1^,^2^,^3^,^4^,^5^,^6^,^7^,^8^. There was a statistically significant but small difference in the overall effect in each outcome that favored trifocal IOLs, with better distance vision compared with bifocal IOLs (MD −0.06; 95% CI, −0.10 to −0.02; *p* = 0.004 for UDVA and MD −0.02; 95% CI, −0.03 to −0.00; *p* = 0.04 for CDVA). The quality of the evidence was low.

Two studies reported uncorrected intermediate visual acuity (UIVA) and distance-corrected intermediate visual acuity (DCIVA) as outcomes (Supplementary Figs S3 and S4)^2^,^6^. There was no significant difference in the overall effect in each outcome (MD −0.10; 95% CI, −0.36 to 0.17; *p* = 0.48 for UIVA and MD −0.12; 95% CI, −0.36 to 0.13; *p* = 0.35 for DCIVA), but only two studies, both characterized by high heterogeneity (I^2^ = 96%, Tau^2^ = 0.03), included this outcome.

Five studies reported uncorrected near visual acuity (UNVA) or distance-corrected near visual acuity (DCNVA) as outcomes (Fig. 2c and d, Table 2, Supplementary Table S3)^2^,^4^,^6^,^7^,^8^. Near VA was not significantly different between the trifocal and bifocal groups (MD −0.01; 95% CI, −0.07 to 0.04; *p* = 0.68 for UNVA and MD −0.01; 95% CI, −0.06 to 0.04; *p* = 0.66 for DCNVA). The quality of the evidence was low. However, the studies were characterized by high heterogeneity (I^2^ > 80%, Tau^2^ = 0.00). One trial (Mojzis *et al*.) had significant problems with comparability and we repeated relevant analysis excluding this trial^6^. Sensitivity analysis revealed Mojzis *et al*.’s study^6^ as the source of statistical heterogeneity for both the UNVA and DCNVA outcomes. After excluding Mojzis *et al*.’s study, no evidence of heterogeneity was detected (I^2^ = 41% for UNVA and I^2^ = 0% for DCNVA), but the results of the previous analysis did not change, as there was still no significant difference between the two groups.

#### Defocus curve

Similar defocus curves were recorded by seven studies (Table 3)^1^,^2^,^3^,^4^,^5^,^6^,^7^, all of which suggested that the trifocal group tended to perform better than the bifocal group, especially at the intermediate distance, although both groups demonstrated a decline in VA at that distance. However, two studies reported that the bifocal group achieved significantly better near VA than the trifocal group^2^,^6^. Among the seven studies, three provided distance-corrected defocus curve data for meta-analyses (Table 4)^1^,^5^,^6^. The results demonstrated that the trifocal group achieved better VA at defocus levels of −1.50 D to −0.50 D than the bifocal group, including a significant difference at intermediate vision (−1.50 D) with low heterogeneity (I^2^ = 23%).

#### Contrast sensitivity

Five studies assessed contrast sensitivity (Table 5)^2^,^3^,^4^,^6^,^8^. Findings differed between photopic and mesopic light conditions. Under photopic light conditions, Cochener^4^ and Jonker *et al*.^2^ reported that no statistically significant differences were observed between the two groups. However, a significantly higher level of contrast sensitivity was found for spatial frequency of three cpd in the trifocal group than in the bifocal group in Mojzis *et al*.’s study^6^. Under mesopic light conditions, Bilbao-Calabuig *et al*.^3^ and Plaza-Puche *et al*.^8^ reported no statistically significant differences between the two groups, but contrast sensitivity values were significantly better in the bifocal group at a frequency of six cpd under mesopic conditions in Jonker *et al*.’s study^2^.

#### Quality of vision

Visual quality as recorded by validated questionnaires was assessed in four studies (Table 6)^1^,^2^,^4^,^5^. High satisfaction was reported in both groups^1^,^4^. No statistically significant differences with respect to subjective visual quality, such as glare or halos, between the trifocal and bifocal groups were reported in most questionnaires^2^,^4^,^5^. However, Gundersen and Potvin^1^ reported that significantly fewer visual disturbances were present in the bifocal group. Spectacle independence was achieved more frequently with trifocal than with bifocal IOL implants in Jonker *et al*.’s study^2^.

#### Refraction

Postoperative refraction—cylinder, sphere, and spherical equivalent—were reported in six studies (Supplementary Figs S5–S7)^1^,^2^,^5^,^6^,^7^,^8^. This meta-analysis did not find any statistically significant difference with respect to postoperative refraction between the trifocal and bifocal groups (MD 0.09; 95% CI, −0.05 to 0.23; *p* = 0.20 for cylinder, MD 0.12; 95% CI, −0.13 to 0.37; *p* = 0.34 for sphere and MD 0.03; 95% CI, −0.06 to 0.13; *p* = 0.47 for spherical equivalent).

### Publication bias

Publication bias was tested using Begg’s and Egger’s tests. These tests did not show significant results in all comparisons (Table 4 and Supplementary Table S3). These results indicated little publication bias.

## Discussion

The present study analyzed whether and to what extent trifocal IOLs perform better than bifocal IOLs in terms of VA (including defocus curve), refraction, contrast sensitivity, and visual quality. The studies were similar in finding a better distance-corrected intermediate (as demonstrated by the defocus curves) VA with trifocal IOLs. However, there was no significant difference in distance VA, near VA or refraction between the two groups. Contrast sensitivity and subjective visual quality yielded less conclusive results.

The variation in follow-up intervals was a major difficulty in conducting this meta-analysis. Subgroup analysis with regard to follow-up length could not be performed due to the limited number of included studies. There is no general accepted follow-up duration for reporting the results of trials involving cataract surgeries. Based on previous studies and the authors’ own clinical experience, data for VA, refraction, contrast sensitivity, and subjective visual quality appears to remain stable at three months postoperatively and beyond, so we pooled the data reported at the end of follow-up for comparison. The combination of data from RCTs and cohort studies was another difficulty. We chose the results of analyses including only RCTs as the primary results for distance VA outcomes. Moreover, there were only one or two RCTs reporting the other outcomes, such as intermediate VA, near VA, refraction, and defocus curve. No heterogeneity between RCTs and cohorts was detected for those outcomes. Considering the inadequate number of RCTs and the high quality of cohorts, we retained cohorts to supplement existing randomized trial evidence.

Data from RCTs on distance VA did not reveal any significant difference between trifocal and bifocal IOLs. Combined with cohorts, the results demonstrated that trifocal IOL implantation provided a statistically significant but small advantage in UDVA and CDVA. The results confirmed that the generation of a third focal point by trifocal IOLs was not detrimental to the distance focal point^6^. The variability between studies in terms of sample size, clinical protocols used to obtain VA measurements, or patient features may have played a major role in the discrepancies among the studies^6^.

In terms of intermediate VA, the present study’s result would not be credible due to the limited number of studies available and their high heterogeneity. Combined with the results of the defocus curve, the trifocal group performed better than the bifocal group at the intermediate distance as expected, since bifocal IOLs have a greater decline in VA in the intermediate range. The meta results of the defocus curve demonstrated that the trifocal group achieved better VA at defocus levels of −1.50 D to −0.50 D than the bifocal group, including a significant but relatively small difference (−0.07) at −1.50 D (intermediate vision) with low heterogeneity (I^2^ = 23%), and small (−0.06) and modest (−0.12) differences with high heterogeneity (I^2^ = 76%) at −0.50 D and −1.00 D, respectively. Since all studies indicated that the trifocal group achieved better intermediate VA than the bifocal group^1^,^2^,^3^,^4^,^5^,^6^,^7^, there was reason to believe that the statistically significant difference was clinically meaningful. Previous bench studies comparing the multifocal components of both IOLs found that trifocal IOLs provide a true third intermediate focal point not found with bifocal IOLs^9^,^10^. Considering that no significant differences were present between the two groups in refraction outcomes, IOL optical behavior appears to be the main factor for this finding^6^. The defocus curve outcome also demonstrated that the trifocal group provided a continuum of functional VA at all three distance ranges. The intermediate focal point and continuum of VA are expected to improve patient satisfaction relative to bifocal IOLs, since bifocal IOLs have a greater decline in VA in the intermediate range^11^.

In terms of near VA, no significant differences were found between the two groups. It is worth noting that the additional intermediate focal point in trifocal IOLs did not appear to impact distance or near vision negatively^5^. However, the quality of evidence supporting the near VA is deemed to be low because of the study design and high heterogeneity. Thus, the conclusion should be interpreted cautiously. Mojzis *et al*.’s study^6^ with lower methodological quality was the source of statistical heterogeneity for the outcome. This study scored zero in comparability part of NOS scale, because it was the only one in which both the most important factor (preoperative VA) and the second important factor (age factor) differed significantly between groups. However, excluding this study did not change the effect estimates.

To understand the visual quality obtained with such multifocal IOLs, it is important to analyze more than just high-contrast VA and refraction. Decreases in contrast sensitivity are reported to be less satisfactory with multifocal IOLs than with monofocal IOLs^8^,^12^,^13^. There is always some concern that the additional focal point provided by a trifocal IOL may reduce contrast sensitivity more than bifocal IOLs by splitting light into three foci^9^. However, our findings show that contrast sensitivity was unlikely to be more problematic with trifocal IOLs. A possible explanation is that a relatively small percentage of energy is dedicated to intermediate vision, as compared to distance and near vision^14^,^15^.

With regard to subjective visual quality, visual function questionnaires such as the Visual Function Index-14 (VF-14) and National Eye Institute Visual Function Questionnaire-39 (NEI VFQ-39) have been adopted. The scores are high for all implants, suggesting satisfactory postoperative vision in both bifocal and trifocal IOLs. Gundersen and Potvin reported that fewer participants with bifocal IOLs experienced bothersome visual disturbances^1^. The authors explained that more participants in the trifocal group were younger than in the bifocal group, and younger people may have relatively higher visual demands or expectations. In any case, most studies reported that any such phenomena were acceptable. It is believed that visual disturbances with trifocal IOLs are minimized due to their smooth surface^16^. The results also showed a high level of spectacle independence and high rates of patient satisfaction in both patient groups^1^,^2^,^4^, especially in the trifocal group^2^.

To our knowledge, no previous systematic review and meta-analysis has been applied to compare trifocal with bifocal IOLs. Considering the various choices between and rapid development in IOL designs, the present study has provided useful guidelines when choosing an IOL is an option. However, the results of this study should be interpreted in the context of several important limitations. First, all of the trials were English-language studies from Europe, so the results may not be generalized to other parts of the world. Second, two studies^1^,^2^ received grants from Alcon Laboratories (Fort Worth, TX, USA), while another study^5^ was funded by FineVision (Liège, Belgium). However, the data extracted from these studies did not reveal any preference for any corporate connections. Finally, the quality assessment performed showed excellent quality for all of the included nonrandomized studies, but the RCTs were of low quality, so more studies, especially high-quality and adequately powered RCTs, are warranted.

In conclusion, good evidence exists that the use of the trifocal IOLs improves distance-corrected intermediate VA without negatively impacting distance or near VA, compared to bifocal IOLs. Contrast sensitivity and subjective visual quality were heterogeneous with no clear results favoring either option.

## Materials and Methods

The systematic review and meta-analysis were performed without language or date restriction and reported according to the Meta-Analysis of Observational Studies in Epidemiology (MOOSE) and Preferred Reporting Items for Systematic Reviews and Meta-Analyses (PRISMA) guidelines^17^,^18^.

### Search strategy

Two reviewers (X.L. and Y.Z.) independently searched the Cochrane Central Register of Controlled Trials (CENTRAL), PubMed, EMBASE, and Web of Science databases, using the following search terms as keywords: trifocal (trifocal, three foci), bifocal (bifocal, two foci), intraocular lens, and cataract. Supplementary Data S1 shows the PubMed search process, updated through November 4, 2016. Two reviewers (X.L. and Y.Z.) then independently screened the titles and abstracts, after which potentially relevant trials were closely analyzed as full manuscripts. Disagreement between the two reviewers was resolved initially by discussion; if agreement could not be reached, a third reviewer (Z.S.) was consulted.

### Eligibility criteria and outcome variables

We strove to include in this study all RCTs and prospective cohorts comparing trifocal and bifocal IOLs in which adult participants were undergoing cataract surgery and multifocal IOL implantation or refractive lens exchanges in one or both eyes. Participants with localized ocular disease like corneal opacities, macular disease, and optic neuropathies were excluded. When multiple trials were reported by the same team from the same institution, only the most complete data set was included. As a further filter, inclusion demanded that studies provide quantified data using continuous variables with means and standard deviations. Study authors were contacted to provide sufficient information when necessary; four authors were contacted and two responded^1^,^5^,^6^.

The primary outcomes were defined as uncorrected VA and corrected VA at near, intermediate, and far distances, defocus curves, contrast sensitivity, and subjective perception of quality of vision. The VA measurements were included in the logarithm of the minimum angle of resolution (logMAR) scale, on which lower scores indicate better vision. The defocus curve is a universally accepted measure for evaluating the range of functional vision at all distances under standard testing conditions after implantation of multifocal IOLs^7^. Three important defocus levels define the most important viewing distances for tasks found in different parts of daily life: 0.00 D, corresponding to distance vision; −1.50 D, corresponding to intermediate vision; and −2.50 D, corresponding to near vision^7^. Contrast sensitivity values of 1.5, 3, 6, 12, and 18 cycles per degree (cpd) in sine-wave mode were included in the analysis. Because contrast sensitivity data was not present in all studies and the specific questions about patients’ subjective visual quality perception differed, these outcomes could not be combined in a meta-analysis. Instead, they are reported descriptively.

Postoperative refraction was defined as a secondary outcome. We recorded postoperative spherical equivalents, sphere, and cylinder in diopters for both trifocal and bifocal groups, conducting a meta-analysis on these results.

### Data collection

Using a standard form, two reviewers (X.L. and Y.Z.) independently extracted study characteristics data and outcome measures. All data collection was double- checked, with discrepancies resolved by discussion.

### Assessment of study quality

The quality of RCTs was assessed using the Jadad scale^19^; cohort quality was assessed on the NOS^20^. The Jadad scale uses the three primary domains of randomization, blinding, and participant dropout. Appropriate randomization and blinding each scored two points, with total scores ranging from zero to five. Studies scoring fewer than three points were considered to be of low quality. The maximum NOS score is nine, based on the assessment of three areas: selection quality (four points maximum), comparability (two points maximum), and outcome measures (three points maximum). Higher score indicated higher study quality. The overall quality of evidence was evaluated using the GRADE working group framework.

### Statistical analysis

Data was analyzed with RevMan software (version 5.3; Cochrane Collaboration, Oxford, United Kingdom). Mean differences (MDs) with 95% confidence intervals (CIs) were calculated for the continuous measures; statistical significance in the level of difference was defined as *p* < 0.05. Forest plots were used to present the results, with lines representing the estimates from different studies and their CIs and boxes graphically representing the weight given to each study in calculating the pooled estimate for a given outcome^21^.

Substantial heterogeneity was detected when I^2^ was >50% or the *p*-value for heterogeneity was <0.10. Publication bias was measured using a Begg funnel plot^22^. The fixed effect model was used when no heterogeneity was observed throughout the studies that were included; otherwise, the random effect model was used^23^,^24^. Sensitivity analysis was performed to determine the influence of a single article on the overall pooled analysis.

## Additional Information

**How to cite this article**: Shen, Z. *et al*. Clinical comparison of patient outcomes following implantation of trifocal or bifocal intraocular lenses: a systematic review and meta-analysis. *Sci. Rep.* **7**, 45337; doi: 10.1038/srep45337 (2017).

**Publisher's note:** Springer Nature remains neutral with regard to jurisdictional claims in published maps and institutional affiliations.

## Supplementary Material

Supplementary Data and Tables

## Figures and Tables

**Table 1:** Characteristics of Studies Included in the Meta-analysis Comparing the trifocal IOLs and bifocal IOLs.

Study	Year	Design	Location	Language	Trifocal IOL				Bifocal IOL				Follow-up (mo)
					Eyes (n)	Patients (n)	Age (yrs)	IOL types	Eyes (n)	Patients (n)	Age (yrs)	IOL types	
Bilbao-Calabuig *et al*.^3^	2015	Randomized	Spain	English	24	12	56.3 ± 6.9	FineVision Micro F	22	11	*56.3 ± 6.9	ReSTOR +2.5/+3.0D (SV6AD2/ SN6AD1)	3
Cochener^4^	2016	Randomized	France	English	30	15	NR	FineVision Micro F	24	12	NR	Tecnis ZMB00	6
Gundersen and Potvin-1^1^	2016	Cohort (prospective)	Norway	English	50	25	53 ± 8	AT Lisa tri 839MP	60	30	65 ± 9	ReSTOR +2.5/+3.0D (SV25T0/SN6AD1)	24
Gundersen and Potvin-2^5^	2016	Randomized	Norway	English	22	11	62.1 ± 7.5	FineVision POD FT (toric)	22	11	70.2 ± 7.8	ReSTOR SND1T (toric)	3
Jonker *et al*.^2^	2015	Randomized	The Netherlands	English	29	15	62.6 ± 8.7	FineVision Micro F	26	13	64.0 ± 8.8	ReSTOR +3.0D (SN6AD1)	6
Mojzis *et al*.^6^	2014	Cohort (prospective)	The Czech Republic	English	30	15	55.2 ± 7.0	AT Lisa tri 839MP	30	15	62.3 ± 5.7	AT Lisa 801	3
Plaza-Puche and Alio^7^	2016	Cohort (prospective)	Spain	English	30	15	66.78 ± 6.20	FineVision Micro F	30	15	62.15 ± 10.27	ReSTOR +3.0D (SN6AD1)	3
Plaza-Puche *et al*.^8^	2016	Cohort (prospective)	Spain	English	30	15	63.00 ± 19.00	AT Lisa tri 839MP	30	15	61.00 ± 14.50	Acri Lisa 366D	3
Totals					255	128			234	119			

IOL = intraocular lens, NR = not reported. *The mean age of trifocal and bifocal groups, no separate data provided.

**Table 2:** Summary of Findings: Comparison between Trifocal IOL and Bifocal IOL.

Outcome	№ of trials	Anticipated absolute effects (95% CI)		№ of eyes (studies)	Quality of the evidence (GRADE)	Comments
		Risk with Bifocal IOL	Risk with Trifocal IOL			
UDVA (only for RCTs)	3	The mean UDVA for RCTs comparing trifocal IOL with bifocal IOL was 0	The mean UDVA for RCTs comparing trifocal IOL with bifocal IOL in the intervention group was 0.03 lower (0.06 lower to 0.01 higher)	153 (3 RCTs)	⊕⊕⊕◯ MODERATE^1^	153 eyes
CDVA (only for RCTs)	4	The mean CDVA for RCTs comparing trifocal IOL with bifocal IOL was 0	The mean CDVA for RCTs comparing trifocal IOL with bifocal IOL in the intervention group was 0 (0.01 fewer to 0.01 higher)	199 (4 RCTs)	⊕⊕⊕⊕ HIGH^1^	199 eyes
UNVA	5	The mean UNVA comparing trifocal IOL with bifocal IOL was 0	The mean UNVA comparing trifocal IOL with bifocal IOL in the intervention group was 0.01 lower (0.07 lower to 0.04 higher)	289 (5 cohorts)	⊕◯◯◯ VERY LOW^2,3^	I^2^ = 86%
DCNVA	5	The mean DCNVA comparing trifocal IOL with bifocal IOL was 0	The mean DCNVA comparing trifocal IOL with bifocal IOL in the intervention group was 0.01 lower (0.06 lower to 0.04 higher)	289 (5 cohorts)^1^	⊕◯◯◯ VERY LOW^2,3^	I^2^ = 86%
Defocus Curve	3	—	—	214 (3 cohorts)^1^	⊕◯◯◯ VERY LOW^2,3^	I^2^ = 0% to 90%

*The risk in the intervention group (and its 95% confidence interval) is based on the assumed risk in the comparison group and the relative effect of the intervention (and its 95% CI).

CI = Confidence interval; MD = Mean difference; I^2^ = extent of inconsistency; RCTs = randomized controlled trials; IOL = intraocular lens; UDVA = uncorrected distance visual acuity; CDVA = corrected distance visual acuity; UNVA = uncorrected near visual acuity; DCNVA = distance-corrected near visual acuity.

^1^Few participants.

^2^Study design is the main reason to downgrade the overall quality of evidence.

^3^High heterogeneity.

**Table 3:** Summary of Defocus Curve.

Study (Year)	Trifocal IOL	Bifocal IOL	Results
Bilbao-Calabuig *et al*.^3^	FineVision Micro F	ReSTOR SV6AD2/ SN6AD1	The trifocal group performed better than the bifocal group in near and intermediate vision at −1.00, −2.00, −2.50, −3.00 and −3.50 D (*p* < 0.05).
Cochener^4^	FineVision Micro F	Tecnis ZMB00	The trifocal group performed better than the bifocal group at −1.00, −1.50, −2.00 and −2.50 D (*p* < 0.05). Although both groups demonstrated a decline in visual acuity (VA) at the intermediate distance.
Gundersen and Potvin-1^1^	AT Lisa tri 839 MP	ReSTOR SV25T0/ SN6AD1	The trifocal group provided better VA at −0.50, −1.00, −1.50 (corresponding to viewing distances from 2 m to 67 cm) and −3.00 D (corresponding to a 33 cm viewing distance) (*p* < 0.05).
Gundersen and Potvin-2^5^	FineVision POD FT	ReSTOR SND1T	Results were not statistically significantly different at any distances except +2.00 (not clinically relevant) and −1.50 D (corresponding to a 67 cm viewing distance).
Jonker *et al*.^2^	FineVision Micro F	ReSTOR SN6AD1	Statistically significantly better VA was present in the trifocal group for the defocus level −1.00 and +1.00 D (*p* < 0.05). And better VA was present in the bifocal group at −5.00, −4.50 and −4.00 D (*p* < 0.05).
Mojzis *et al*.^6^	AT Lisa tri 839 MP	AT Lisa 801	The VA was significantly better in the trifocal group compared to the bifocal group for the defocus levels of −0.50, −1.00 and −1.50 D (*p* < 0.05). And better VA was present in the bifocal group at −3.50 and −4.00 D (*p* < 0.05).
Plaza-Puche and Alio^7^	FineVision Micro F	ReSTOR SN6AD1	Statistically significant better VA for defocus levels of −1.50 and −1.00 D was present in the trifocal group.

IOL = intraocular lens. VA = visual acuity. 0.00 D = distance vision, −1.50 D = intermediate vision and −2.50 D = near vision.

**Table 4:** Results of Meta-analyses for Defocus Curve.

Defocus levels (D)	MD (95% CI)	P value	Heterogeneity		Publication bias	
			I^2^	P_heterogeneity_	Begg	Egger
+1.00	−0.03 [−0.06, 0.00]	0.08	10%	0.33	1.000	0.683
+0.50	0.01 [−0.01, 0.03]	0.35	39%	0.19	1.000	0.801
0.00	−0.00 [−0.02, 0.01]	0.60	47%	0.15	1.000	0.820
−0.50	−0.06 [−0.10, −0.01]	0.01	76%	0.02	0.296	0.630
−1.00	−0.12 [−0.18, −0.07]	<0.0001	76%	0.02	1.000	0.996
−1.50	−0.07 [−0.10, −0.05]	<0.00001	23%	0.27	0.296	0.121
−2.00	−0.01 [−0.06, 0.04]	0.76	68%	0.05	1.000	0.607
−2.50	−0.02 [−0.04, 0.01]	0.18	0%	0.81	1.000	0.355
−3.00	−0.03 [−0.12, 0.06]	0.53	82%	0.004	1.000	0.498
−3.50	0.01 [−0.13, 0.14]	0.92	90%	<0.0001	1.000	0.724
−4.00	0.03 [−0.09, 0.16]	0.59	88%	0.0003	1.000	0.801

MD = mean difference, CI = confidence interval, I^2^ = extent of inconsistency. 0.00 D = distance vision, −1.50 D = intermediate vision and −2.50 D = near vision.

**Table 5:** Summary of Contrast Sensitivity.

Study (Year)	Trifocal IOL	Bifocal IOL	Results
**Under photopic light conditions**			
Cochener^4^	FineVision Micro F	Tecnis ZMB00	No significant differences in contrast sensitivity were found between groups.
Jonker *et al*.^2^	FineVision Micro F	ReSTOR SN6AD1	No significant differences in contrast sensitivity were found between groups.
Mojzis *et al*.^6^	AT Lisa tri 839MP	AT Lisa 801	A significantly higher level of contrast sensitivity was found for 3 cpd in the trifocal group compared to the bifocal group.
**Under mesopic light conditions**			
Bilbao-Calabuig *et al*.^3^	FineVision Micro F	ReSTOR SV6AD2/ SN6AD1	No significant differences in contrast sensitivity were found between groups.
Jonker *et al*.^2^	FineVision Micro F	ReSTOR SN6AD1	A significantly higher level of contrast sensitivity was found for 6 cpd in the bifocal group compared to the trifocal group.
Plaza-Puche *et al*.^8^	AT Lisa tri 839MP	Acri Lisa 366D	No significant differences in contrast sensitivity were found between groups.

IOL = intraocular lens.

**Table 6:** Summary of Quality of Vision as Reported in Validated Questionnaires.

Study (Year)	Trifocal IOL	Bifocal IOL	Questionnaire		Results		
Cochener^4^	FineVision Micro F	Tecnis ZMB00	VF-14		Trifocal group	Bifocal group	*p*
				Spectacle independence	100%	92%	0.90
				Halos	92%	67%	0.20
				Glare	58%	50%	0.60
				General satisfaction	93%	92%	0.80
Gundersen and Potvin-1^1^	AT Lisa tri 839MP	ReSTOR SV25T0/SN6AD1	● NEI VFQ-39 ● Quality of Vision	NEI VFQ-39 questionnaire: Both groups had scores over 90; there was no significant difference in scores by group (*p* = 0.25).			
				Quality of Vision survey: There was no significant difference between groups in frequency (*p* = 0.72), severity (*p* = 0.51) or bothersome (*p* = 0.26). 68% of the trifocal subjects and 90% of the bifocal subjects rated visual disturbances as 0 (*p* = 0.045).			
Gundersen and Potvin-2^5^	FineVision POD FT	ReSTOR SND1T	NEI VFQ-25	There was no significant differences between groups (*p* > 0.26).			
Jonker *et al*.^2^	FineVision Micro F	ReSTOR SN6AD1	NEI-RQL 42	The occurrence of side effects, such as glare and halos, was similar in both groups. Twelve (80%) and six (50%) patients reported complete spectacle independence in the trifocal and bifocal groups, respectively.			

IOL = intraocular lens; VF-14 = Visual Function Index-14; NEI VFQ = National Eye Institute Visual Function Questionnaire; NEI-RQL 42 = National Eye Institute Refractive Error Correction Quality of Life Instrument-42.
