# Supplementary material for: Clinical comparison of patient outcomes following implantation of trifocal or bifocal intraocular lenses: a systematic review and meta-analysis
Source: Sci Rep. 2017 Mar 28;7:45337. doi: 10.1038/srep45337 (PMC5368599; doi:10.1038/srep45337)
Supplement: Supplementary Data and Tables [file srep45337-s1.pdf]

Title Page

Full title: Clinical comparison of patient outcomes following implantation of trifocal or bifocal intraocular lenses: a systematic review and meta-analysis

Authors. Zeren Shen[1], Yuchen Lin[1], Yanan Zhu[1], Xin Liu[1], Jie Yan[1] & Ke Yao[1,*]

Author information

[1]Eye Center, Second Affiliated Hospital, School of Medicine, Zhejiang University, Hangzhou, China

*Correspondence: Ke YAO, MD, Professor and Chief. Eye Center, Second Affiliated Hospital, School of Medicine, Zhejiang University, No. 88 Jiefang Road, Hangzhou 310009, China. E-mail address: xlren@zju.edu.cn.

Supplementary Information

Supplementary Data S1. Search strategy of PubMed.

Supplementary Table S1. Jadad Scale for randomized controlled trials (RCTs).

Supplementary Table S2. Newcastle–Ottawa Scale for observational studies (cohorts).

Supplementary Table S3. Pooled MD, heterogeneity and publication Bias.

Supplementary Fig. S1. Forest plot showing the mean difference (MD) of uncorrected distance visual acuity (UDVA; logMAR) comparing trifocal intraocular lens (IOL) with bifocal IOL postoperatively.

Supplementary Fig. S2. Forest plot showing the mean difference (MD) of corrected distance visual acuity (CDVA; logMAR) comparing trifocal intraocular lens (IOL) with bifocal IOL postoperatively.

Supplementary Fig. S3. Forest plot showing the mean difference (MD) of uncorrected intermediate visual acuity (UIVA; logMAR) comparing trifocal intraocular lens (IOL) with bifocal IOL postoperatively.

Supplementary Fig. S4. Forest plot showing the mean difference (MD) of distance-corrected intermediate visual acuity (DCIVA; logMAR) comparing trifocal intraocular lens (IOL) with bifocal IOL postoperatively.

Supplementary Fig. S5. Forest plot showing the mean difference (MD) of postoperative cylinder (D) comparing trifocal intraocular lens (IOL) with bifocal IOL.

Supplementary Fig. S6. Forest plot showing the mean difference (MD) of postoperative sphere (D) comparing trifocal intraocular lens (IOL) with bifocal IOL.

Supplementary Fig. S7. Forest plot showing the mean difference (MD) of postoperative spherical equivalent (SE; D) comparing trifocal intraocular lens (IOL) with bifocal IOL.

Supplementary Data

Supplementary Data S1. Medline (PubMed) search strategy

#1 Cataract[Mesh]

#2 cataract*[tiab]

#3 pseudoaphakia*[tiab]

#4 phakectom*[tiab]

#5 #1 OR #2 OR #3 OR #4

#6 Lenses, Intraocular[Mesh]

#7 lens*[tiab]

#8 IOL*[tiab]

#9 #6 OR #7 OR #8

#10 trifocal*[tiab]

#11 three foci[tiab]

#12 3 foci[tiab]

#13 #10 OR #11 OR #12

#14 bifocal*[tiab]

#15 two foci[tiab]

#16 2 foci[tiab]

#17 #14 OR #15 OR #16

#18 #5 AND #9 AND #13 AND #17

Supplementary Tables

Supplementary Table S1. Jadad Scale for Randomized Controlled Trials (RCTs)

Study	Randomization	Blinding	Withdraws	Sum of Score
Bilbao-Calabuig et al[3]	1	0	0	1
Cochener[4]	1	0	1	2
Gundersen and Potvin-2[5]	1	0	1	2
Jonker et al[2]	1	1	1	3

A higher overall score corresponds to a lower risk of bias; a score of three or less (out of five) indicates a high risk of bias.

Supplementary Table S2. Newcastle–Ottawa Scale for Observational Studies (cohorts)

Study	Selection	Comparability	Outcome	Total score
Gundersen and Potvin-1[1]	****		***	7
Mojzis et al[6]	****		***	7
Plaza-Puche and Alio[7]	****	**	**	8
Plaza-Puche et al[8]	****	*	**	7

A higher overall score corresponds to a lower risk of bias; a score of six or more (out of nine) indicates a low risk of bias. Each * equals 1 point.

Supplementary Table S3. Pooled MD, Heterogeneity and Publication Bias in Meta-analysis: Comparison between Trifocal IOL and Bifocal IOL

Outcome	№ of trials	MD (95% CI)	P value	Heterogeneity		Publication bias	
				I^2	$P_{heterogeneity}$	Begg	Egger
UDVA (only for RCTs)	3	-0.03 [-0.06, 0.01]	0.13	0%	0.48	1.000	0.808
CDVA (only for RCTs)	4	-0.00 [-0.01, 0.01]	0.78	0%	0.77	0.308	0.214
UNVA	5	-0.01 [-0.07, 0.04]	0.68	86%	< 0.0001	0.462	0.938
DCNVA	5	-0.01 [-0.06, 0.04]	0.66	86%	< 0.0001	0.806	0.564

MD = mean difference; CI = confidence interval; I^2: extent of inconsistency; RCT = randomized controlled trial; IOL = intraocular lens; UDVA = uncorrected distance visual acuity; CDVA = corrected distance visual acuity; UNVA = uncorrected near visual acuity; DCNVA = distance-corrected near visual acuity.

Supplementary Figures

Study or Subgroup	Trifocal Mean	Trifocal SD	Trifocal Total	Bifocal Mean	Bifocal SD	Bifocal Total	Weight	Mean Difference IV, Random, 95% CI
Cochener 2016	0.08	0.08	30	0.11	0.09	24	19.4%	−0.03 [−0.08, 0.02]
Gundersen−2 2016	0.03	0.1	22	0.08	0.13	22	14.8%	−0.05 [−0.12, 0.02]
Jonker 2015	0.09	0.16	29	0.08	0.11	26	14.2%	0.01 [−0.06, 0.08]
Mojzis 2014	−0.05	0.08	30	0	0.13	30	17.6%	−0.05 [−0.10, 0.00]
Plaza−Puche−1 2016	0.1	0.09	30	0.19	0.14	30	16.6%	−0.09 [−0.15, −0.03]
Plaza−Puche−2 2016	−0.1	0.1	30	0.04	0.12	30	17.3%	−0.14 [−0.20, −0.08]
**Total (95% CI)**			**171**			**162**	**100.0%**	**−0.06 [−0.10, −0.02]**

Heterogeneity: $Tau^2 = 0.00$; $Chi^2 = 14.32$, df = 5 (P = 0.01); $I^2 = 65\%$
Test for overall effect: Z = 2.90 (P = 0.004)

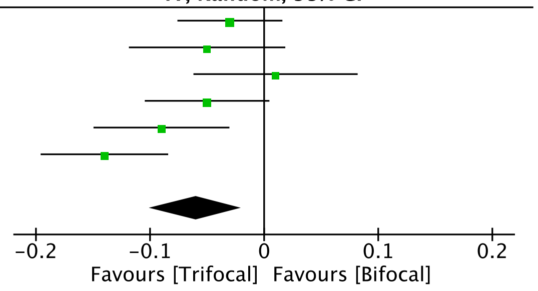

Supplementary Fig. S1. Forest plot showing the mean difference (MD) of uncorrected distance visual acuity (UDVA; logMAR) comparing trifocal intraocular lens (IOL) with bifocal IOL postoperatively.

Study or Subgroup	Trifocal Mean	Trifocal SD	Trifocal Total	Bifocal Mean	Bifocal SD	Bifocal Total	Weight	Mean Difference IV, Random, 95% CI
Bilbao−Calabuig 2016	0.01	0.02	24	0.01	0.01	22	20.8%	0.00 [−0.01, 0.01]
Cochener 2016	0.03	0.04	30	0.03	0.04	24	15.9%	0.00 [−0.02, 0.02]
Gundersen−1 2016	−0.03	0.04	50	−0.02	0.05	60	17.9%	−0.01 [−0.03, 0.01]
Gundersen−2 2016	−0.01	0.06	22	0.01	0.07	22	9.8%	−0.02 [−0.06, 0.02]
Jonker 2015	0.01	0.11	29	0.02	0.08	26	7.0%	−0.01 [−0.06, 0.04]
Mojzis 2014	−0.06	0.07	30	−0.03	0.11	30	7.8%	−0.03 [−0.08, 0.02]
Plaza−Puche−1 2016	0.05	0.07	30	0.06	0.05	30	12.3%	−0.01 [−0.04, 0.02]
Plaza−Puche−2 2016	−0.1	0.12	30	0	0.001	30	8.6%	−0.10 [−0.14, −0.06]
**Total (95% CI)**			**245**			**244**	**100.0%**	**−0.02 [−0.03, −0.00]**

Heterogeneity: $Tau^2 = 0.00$; $Chi^2 = 22.23$, df = 7 (P = 0.002); $I^2 = 69\%$
Test for overall effect: Z = 2.02 (P = 0.04)

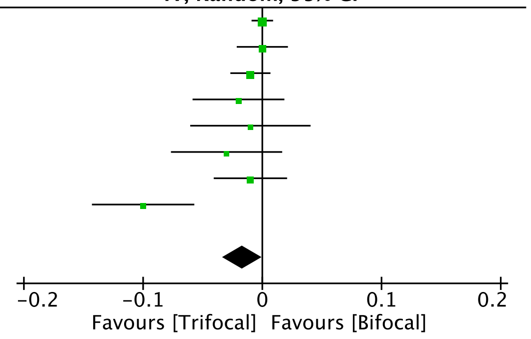

Supplementary Fig. S2. Forest plot showing the mean difference (MD) of corrected distance visual acuity (CDVA; logMAR) comparing trifocal intraocular lens (IOL) with bifocal IOL postoperatively.

Study or Subgroup	Trifocal Mean	Trifocal SD	Trifocal Total	Bifocal Mean	Bifocal SD	Bifocal Total	Weight	Mean Difference IV, Random, 95% CI
Jonker 2015	0.45	0.18	29	0.41	0.15	26	49.5%	0.04 [−0.05, 0.13]
Mojzis 2014	0.06	0.07	30	0.29	0.18	30	50.5%	−0.23 [−0.30, −0.16]
**Total (95% CI)**			**59**			**56**	**100.0%**	**−0.10 [−0.36, 0.17]**

Heterogeneity: $Tau^2 = 0.03$; $Chi^2 = 22.60$, df = 1 (P < 0.00001); $I^2 = 96\%$
Test for overall effect: Z = 0.71 (P = 0.48)

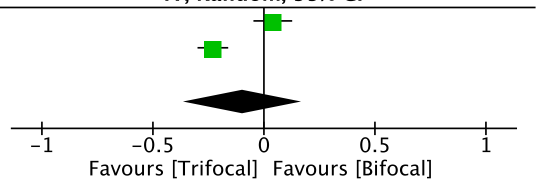

Supplementary Fig. S3. Forest plot showing the mean difference (MD) of uncorrected intermediate visual acuity (UIVA; logMAR) comparing trifocal intraocular lens (IOL) with bifocal IOL postoperatively.

Study or Subgroup	Trifocal Mean	Trifocal SD	Trifocal Total	Bifocal Mean	Bifocal SD	Bifocal Total	Weight	Mean Difference IV, Random, 95% CI
Jonker 2015	0.43	0.15	29	0.42	0.14	26	49.7%	0.01 [−0.07, 0.09]
Mojzis 2014	0.06	0.07	30	0.3	0.17	30	50.3%	−0.24 [−0.31, −0.17]
**Total (95% CI)**			**59**			**56**	**100.0%**	**−0.12 [−0.36, 0.13]**

Heterogeneity: $Tau^2 = 0.03$; $Chi^2 = 23.53$, df = 1 (P < 0.00001); $I^2 = 96\%$
Test for overall effect: Z = 0.93 (P = 0.35)

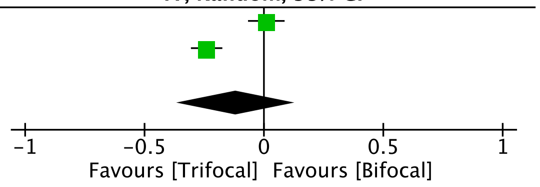

Supplementary Fig. S4. Forest plot showing the mean difference (MD) of distance-corrected intermediate visual acuity (DCIVA; logMAR) comparing trifocal intraocular lens (IOL) with bifocal IOL postoperatively.

Study or Subgroup	trifocal IOL Mean	SD	Total	bifocal IOL Mean	SD	Total	Weight	Mean Difference IV, Random, 95% CI
Gundersen-1 2016	−0.32	0.3	50	−0.4	0.32	60	22.4%	0.08 [−0.04, 0.20]
Gundersen-2 2016	−0.3	0.26	22	−0.49	0.45	22	16.0%	0.19 [−0.03, 0.41]
Jonker 2015	−0.9	0.61	29	−0.66	0.42	26	13.0%	−0.24 [−0.51, 0.03]
Mojzis 2014	−0.2	0.21	30	−0.46	0.33	30	20.9%	0.26 [0.12, 0.40]
Plaza-Puche-1 2016	−0.78	0.42	30	−0.68	0.56	30	14.2%	−0.10 [−0.35, 0.15]
Plaza-Puche-2 2016	−0.25	0.25	30	−0.5	0.69	30	13.6%	0.25 [−0.01, 0.51]
**Total (95% CI)**			**191**			**198**	**100.0%**	**0.09 [−0.05, 0.23]**

Heterogeneity: $Tau^2 = 0.02$; $Chi^2 = 15.20$, df = 5 (P = 0.010); $I^2 = 67\%$
Test for overall effect: Z = 1.29 (P = 0.20)

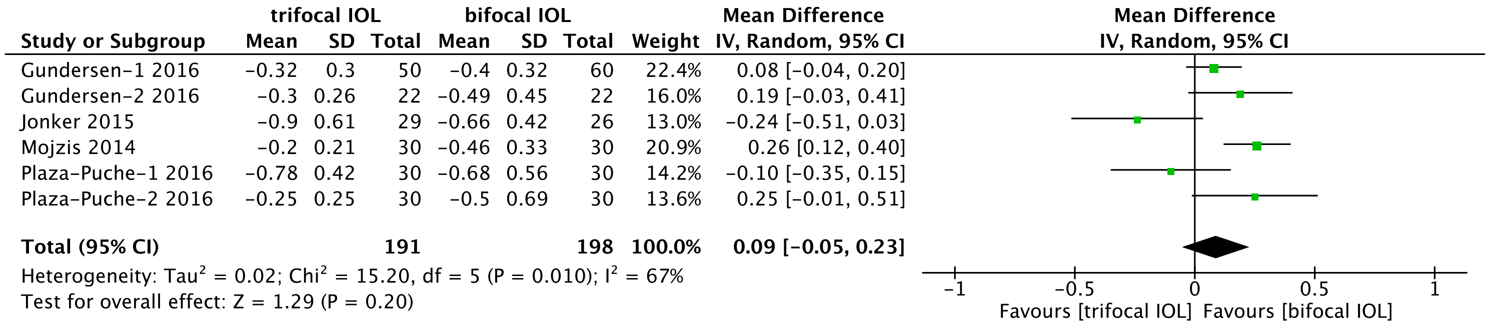

Supplementary Fig. S5. Forest plot showing the mean difference (MD) of postoperative cylinder (D) comparing trifocal intraocular lens (IOL) with bifocal IOL.

Study or Subgroup	trifocal IOL Mean	SD	Total	bifocal IOL Mean	SD	Total	Weight	Mean Difference IV, Random, 95% CI
Mojzis 2014	−0.19	0.35	30	−0.18	0.38	30	36.4%	−0.01 [−0.19, 0.17]
Plaza-Puche-1 2016	0.36	0.5	30	−0.01	0.36	30	33.6%	0.37 [0.15, 0.59]
Plaza-Puche-2 2016	0	0.25	30	0	0.7	30	30.0%	0.00 [−0.27, 0.27]
**Total (95% CI)**			**90**			**90**	**100.0%**	**0.12 [−0.13, 0.37]**

Heterogeneity: $Tau^2 = 0.04$; $Chi^2 = 7.61$, df = 2 (P = 0.02); $I^2 = 74\%$
Test for overall effect: Z = 0.95 (P = 0.34)

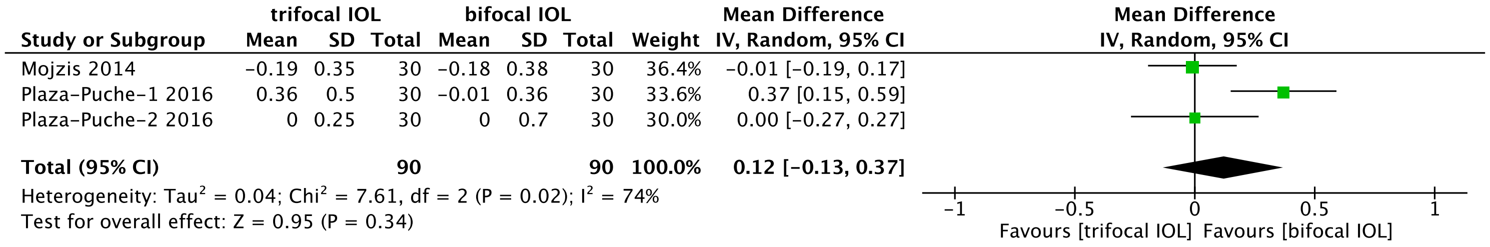

Supplementary Fig. S6. Forest plot showing the mean difference (MD) of postoperative sphere (D) comparing trifocal intraocular lens (IOL) with bifocal IOL.

Study or Subgroup	trifocal IOL Mean	SD	Total	bifocal IOL Mean	SD	Total	Weight	Mean Difference IV, Fixed, 95% CI
Gundersen-1 2016	0.19	0.4	50	0.18	0.32	60	44.0%	0.01 [−0.13, 0.15]
Gundersen-2 2016	0.22	0.38	22	0.09	0.42	22	14.8%	0.13 [−0.11, 0.37]
Jonker 2015	0.03	0.52	29	0.11	0.25	26	18.4%	−0.08 [−0.29, 0.13]
Mojzis 2014	−0.29	0.33	30	−0.4	0.42	30	22.7%	0.11 [−0.08, 0.30]
**Total (95% CI)**			**131**			**138**	**100.0%**	**0.03 [−0.06, 0.13]**

Heterogeneity: $Chi^2 = 2.46$, df = 3 (P = 0.48); $I^2 = 0\%$
Test for overall effect: Z = 0.73 (P = 0.47)

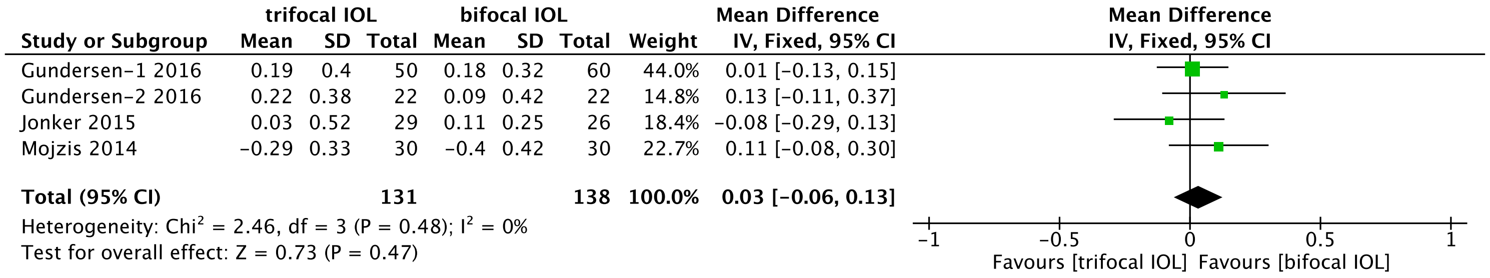

Supplementary Fig. S7. Forest plot showing the mean difference (MD) of postoperative spherical equivalent (SE; D) comparing trifocal intraocular lens (IOL) with bifocal IOL.
